
JOURNAL INFORMATION
==============================
NLM Title Abbreviation: Orthop Surg
Journal ID: Orthop Surg
NLM Journal ID: 101501666
Journal ID: OS

Orthopaedic Surgery

ISSN: 1757-7853
EISSN: 1757-7861
Publisher: Wiley

ARTICLE INFORMATION
==============================
PMCID: PMC11474579
DOI: 10.1111/os.13784
Article ID: OS13784
Article version: 1
Subjects: Issue Information, Issue Information

Issue Information

Electronic publication date: 2024 Apr 1
Publication date: 2024 Apr
Volume: 16
Issue: 4
First page: 789
Last page: 790
Copyright: © 2024 The Authors. Orthopaedic Surgery published by Tianjin Hospital and John Wiley & Sons Australia, Ltd.
License: This is an open access article under the terms of the http://creativecommons.org/licenses/by/4.0/ License, which permits use, distribution and reproduction in any medium, provided the original work is properly cited.
License URL: https://creativecommons.org/licenses/by/4.0/

Figure count: 0
Table count: 0
Page count: 1

source-schema-version-number: 2.0
cover-date: April 2024
details-of-publishers-convertor: Converter:WILEY_ML3GV2_TO_JATSPMC version:6.4.9 mode:remove_FC converted:15.10.2024

==============================

Volume 16 • Issue 4 • April 2024 • ISSN 1757-7861

### Orthopaedic Surgery

Open Access

REVIEW ARTICLES

791
PATIENT-RELATED PREDICTORS FOR THE FUNCTIONAL OUTCOME OF SUPERPATH HEMIARTHROPLASTY VERSUS CONVENTIONAL APPROACH HEMIARTHROPLASTY: A SYSTEMATIC REVIEW AND META-REGRESSION ANALYSIS OF RANDOMIZED CONTROLLED TRIALS

802
HOW DO MUSCLE FUNCTION AND QUALITY AFFECT THE PROGRESSION OF KOA? A NARRATIVE REVIEW

811
BIOMECHANICAL EFFECTS OF MECHANICAL STRESS ON CELLS INVOLVED IN FRACTURE HEALING

CLINICAL ARTICLES

821
3D-PRINTED PERSONALIZED LATTICE IMPLANT AS AN INNOVATIVE STRATEGY TO RECONSTRUCT GEOGRAPHIC DEFECTS IN LOAD-BEARING BONES

830
DISTAL JUNCTIONAL FAILURES IN DEGENERATIVE THORACOLUMBAR HYPERKYPHOSIS

842
RISK FACTORS OF HIDDEN BLOOD LOSS IN UNILATERAL BIPORTAL ENDOSCOPIC SURGERY FOR PATIENTS WITH LUMBAR SPINAL STENOSIS

851
CORRELATION BETWEEN DISC IMAGING OBSERVATIONS AND CLINICAL EFFICACY AFTER PERCUTANEOUS ENDOSCOPIC LUMBAR DISCECTOMY: A 1-YEAR FOLLOW-UP STUDY

864
WHETHER PATIENTS WITH ANTERIOR CRUCIATE LIGAMENT RECONSTRUCTION WALKING AT A FAST SPEED SHOW MORE KINEMATIC ASYMMETRIES?

873
EFFECT OF PERINEURAL OR INTRAVENOUS BETAMETHASONE ON FEMORAL NERVE BLOCK OUTCOMES IN KNEE ARTHROPLASTY: A RANDOMIZED, CONTROLLED STUDY

882
SAFETY AND EFFECTIVENESS OF ROBOTIC-ARM ASSISTED TOTAL KNEE ARTHROPLASTY

894
TOPICAL USE OF LOW-DOSE TRANEXAMIC ACID HAS NO EFFECT ON DRAINAGE VOLUME AFTER MEDIAL OPEN WEDGE HIGH TIBIAL OSTEOTOMY: A CASE CONTROL STUDY

902
NO DIFFERENCE IN BONE TUNNEL ENLARGEMENT AND CLINICAL OUTCOME BETWEEN CORTICAL SUSPENSION AND HYBRID FEMORAL FIXATION IN HAMSTRING ANTERIOR CRUCIATE LIGAMENT RECONSTRUCTION

912
LONG-TERM OUTCOME OF ISOBAR TTL SYSTEM FOR THE TREATMENT OF LUMBAR DEGENERATIVE DISC DISEASES

921
ONE-STAGE NEURORRHAPHY AND POSTERIOR TRANSPOSITION WITH GASTROCNEMIUS FASCIAL FLAP FOR COMMON PERONEAL NERVE INJURY: PRELIMINARY RESULTS

RESEARCH ARTICLES

930
IMPORTANCE OF THE POSTERIOR PLATE IN THREE-COLUMN TIBIAL PLATEAU FRACTURES: A FINITE ELEMENT ANALYSIS AND CLINICAL VALIDATION

943
EVALUATION OF THE ANATOMICAL REFERENCE POINT IN POSTERIOR MINIMALLY INVASIVE ATLANTOAXIAL SPINE SURGERY: A CADAVERIC ANATOMICAL STUDY

953
BIOMECHANICAL RESEARCH OF THREE PARALLEL CANNULATED COMPRESSION SCREWS IN OBLIQUE TRIANGLE CONFIGURATION FOR FIXATION OF FEMORAL NECK UNSTABLE FRACTURES

965
GUT MICROBIOTA ALTERATIONS IN ADOLESCENT IDIOPATHIC SCOLIOSIS ARE ASSOCIATED WITH ABERRANT BONE HOMEOSTASIS

976
DESIGN AND BIOMECHANICAL FINITE ELEMENT ANALYSIS OF SPATIAL WEAVING INFRACALCANEAL FIXATOR SYSTEM

984
THE APPLICATION OF SESAMOID POSITION IN DIAGNOSING THUMB METACARPOPHALANGEAL JOINT DORSAL DISLOCATION: A RETROSPECTIVE STUDY

OPERATIVE TECHNIQUES

989
A COUNTERTRACTION CLOSED REDUCTION TECHNIQUE IN MINIMALLY INVASIVE FIXATION OF RECENT TYPE C PELVIC RING INJURIES

998
ENDOSCOPIC ARTHROPLASTY *VIA* MINI-OPEN DIRECT ANTERIOR APPROACH IMPROVES POSTOPERATIVE COMPLICATIONS AND ACETABULAR COMPONENTS OF TOTAL HIP ARTHROPLASTY IN OBESE PATIENTS

CASE REPORT

1010
REVISION FOR SOLID-BODY BREAKAGE OF THE 3D-PRINTED IMPLANT FOLLOWING JOINT-SPARING SURGERY: A TECHNICAL NOTE

WILEY

View this journal online at wileyonlinelibrary.com/journal/os

### Orthopaedic Surgery

**Honorary Editor-in-Chief**
Guixing Qiu, *Beijing, China*

**Editor-in-Chief**
Xinlong Ma, *Tianjin, China*

**Associate Editors**
Cyrus Cooper, *Southampton, UK*
Benjamin G. Domb, *Des Plaines, USA*
Qing Jiang, *Nanjing, China*
Baoge Liu, *Beijing, China*
Zhuojing Luo, *Xi'an, China*
Luigi Meccariello, *Moiano, Italy*
Ling Qin, *Hong Kong, China*
Qi Yin, *Liverpool, UK*

**Editorial Board**
Elisa Belluzzi, *Padua, Italy*
Carlo Biz, *Padua, Italy*
Mary L Bouxsein, *Boston, USA*
Richard E. Buckley, *Calgary, Canada*
Zhengdong Cai, *Shanghai, China*
Wei Chai, *Beijing, China*
Bohua Chen, *Qingdao, China*
Liang Chen, *Soochow, China*
Yanxi Chen, *Shanghai, China*
Pramod Devkota, *Patan, Nepal*
Meng Fan, *Tianjin, China*
Haoyu Feng, *Taiyuan, China*
Maoqi Gong, *Beijing, China*
Yong Hai, *Beijing, China*
Wei Huang, *Chongqing, China*
Baochao Ji, *Urumqi, China*
Ayhan Kanat, *Rize, Turkey*
Kenny Kwan, *Hong Kong, China*
Sallie Lamb, *Exeter, UK*
Lifeng Lao, *Shanghai, China*
Jung-Hee Lee, *Seoul, Korea*
Guanghua Lei, *Changsha, China*
Guoan Li, *Newton, USA*
Hui Li, *Tianjin, China*
Jianmin Li, *Jinan, China*
Weishi Li, *Beijing, China*
Jianhao Lin, *Beijing, China*
Fanxiao Liu, *Munich, Germany*
Ruben Lostado, *La Rioja, Spain*
Songchen Lyu, *Harbin, China*
Weihu Ma, *Ningbo, China*
Xin Ma, *Shanghai, China*
Masaya Nakamura, *Tokyo, Japan*
Muthu Sathish, *Velayuthampalayam, India*
Wolfgang Senker, *Linz, Austria*
Sancar Serbest, *Boston, USA*
Cailiang Shen, *Hefei, China*
Bin Shen, *Chengdu, China*
Huiyong Shen, *Shenzhen, China*
Jianxiong Shen, *Beijing, China*
Puyi Sheng, *Guangzhou, China*
Junying Sun, *Soochow, China*
Shui Sun, *Jinan, China*
YasuhikoTakegami, *Nagoya, Japan*
Sakae Tanaka, *Tokyo, Japan*
Xiaodong Tang, *Beijing, China*
Fei Wang, *Shijiazhuang, China*
Weijun Wang, *Nanjing, China*
Xu Wang, *Shanghai, China*
Zheng Wang, *Beijing, China*
Jidapa Wongcharoenwatana, *Bangkok, Thailand*
Chi-Chuan Wu, *Taipei, China*
Nan Wu, *Beijing, China*
Zhihong Wu, *Beijing, China*
Yayi Xia, *Lanzhou, China*
Jianru Xiao, *Shanghai, China*
Zhongyu Xie, *Shenzhen, China*
Dan Xing, *Beijing, China*
Weiguo Xu, *Tianjin, China*
Peng Xu, *Xi 'an, China*
CaoYang, *Wuhan, China*
Pei Yang, *Xi 'an, China*
Zhaoming Ye, *Hangzhou, China*
Xiuchun Yu, *Jinan, China*
Bin Yu, *Guangzhou, China*
Yi Zeng, *Chengdu, China*
Dianying Zhang, *Beijing, China*
Hong Zhang, *Texas, USA*
Weibin Zhang, *Shanghai, China*
Xianlong Zhang, *Shanghai, China*
Jianguo Zhang, *Beijing, China*
Bin Zhao, *Taiyuan, China*
Jie Zhao, *Shanghai, China*
Yu Zhao, *Beijing, China*
Zongke Zhou, *Chengdu, China*
Yue Zhu, *Shenyang, China*
Zezhang Zhu, *Nanjing, China*

**Address for Editorial Correspondence:** Editorial Board of *Orthopaedic Surgery*, Tianjin Hospital, 122 Munan Road, Tianjin 300050, China (email: orthopaedicsurgery@126.com; telephone: +86 22 2833 4734; fax: +86 22 2824 1184).

**Disclaimer:** The Publisher, Tianjin Hospital and Editors cannot be held responsible for errors or any consequences arising from the use of information contained in this journal; the views and opinions expressed do not necessarily reflect those of the Publisher, Tianjin Hospital and Editors, neither does the publication of advertisements constitute any endorsement by the Publisher, Tianjin Hospital and Editors of the products advertised.

**Copyright © 2024 The Authors. Orthopaedic Surgery published by Tianjin Hospital and John Wiley & Sons Australia, Ltd**

**Copyright and Copying (in any format)**
Copyright © 2024 The Authors. Orthopaedic Surgery published by Tianjin Hospital and John Wiley & Sons Australia, Ltd. All rights reserved. No part of this publication may be reproduced, stored or transmitted in any form or by any means without the prior permission in writing from the copyright holder. Authorization to copy items for internal and personal use is granted by the copyright holder for libraries and other users registered with their local Reproduction Rights Organisation (RRO), e.g. Copyright Clearance Center (CCC), 222 Rosewood Drive, Danvers, MA 01923, USA (www.copyright.com), provided the appropriate fee is paid directly to the RRO. This consent does not extend to other kinds of copying such as copying for general distribution, for advertising or promotional purposes, for republication, for creating new collective works or for resale. Permissions for such reuse can be obtained using the RightsLink "Request Permissions" link on Wiley Online Library. Special requests should be addressed to: permissions@wiley.com

For submission instructions, subscription and all other information visit: http://wileyonlinelibrary.com/journal/os

ISSN 1757-7861 (Online)

OS.JEB.April24
